# Supplementary material for: Multi-omic and functional analysis for classification and treatment of sarcomas with FUS-TFCP2 or EWSR1-TFCP2 fusions
Source: Nat Commun. 2024 Jan 2;15:51. doi: 10.1038/s41467-023-44360-2 (PMC10761971; doi:10.1038/s41467-023-44360-2)
Supplement: Supplementary file 3 — Description of Additional Supplementary Files [file 41467_2023_44360_MOESM3_ESM.pdf]

Description of Additional Supplementary Files

Supplementary Data 1: Characteristics of patients with FUS/EWSR1-TFCP2 sarcoma

Supplementary Data 2: Differentially expressed genes in FUS/EWSR-TFCP2 sarcoma versus other RMS subtypes

Supplementary Data 3: Differentially expressed genes in MCF10A and SCP-1 cells stably transduced with empty vector (EV), FUS-TFCP2, EWSR1-TFCP2, TFCP2, FUS, or EWSR1
