# Supplementary material for: Multi-omic and functional analysis for classification and treatment of sarcomas with FUS-TFCP2 or EWSR1-TFCP2 fusions
Source: Nat Commun. 2024 Jan 2;15:51. doi: 10.1038/s41467-023-44360-2 (PMC10761971; doi:10.1038/s41467-023-44360-2)
Supplement: Supplementary file 7 — Reporting Summary [file 41467_2023_44360_MOESM7_ESM.pdf]

nature portfolio

Corresponding author(s): Claudia Scholl, Stefan Fröhling

Last updated by author(s): Dec 7, 2023

Reporting Summary

Nature Portfolio wishes to improve the reproducibility of the work that we publish. This form provides structure for consistency and transparency in reporting. For further information on Nature Portfolio policies, see our Editorial Policies and the Editorial Policy Checklist.

Statistics

For all statistical analyses, confirm that the following items are present in the figure legend, table legend, main text, or Methods section.

n/a	Confirmed	
☐	☒	The exact sample size (*n*) for each experimental group/condition, given as a discrete number and unit of measurement
☒	☐	A statement on whether measurements were taken from distinct samples or whether the same sample was measured repeatedly
☐	☒	The statistical test(s) used AND whether they are one- or two-sided<br>*Only common tests should be described solely by name; describe more complex techniques in the Methods section.*
☒	☐	A description of all covariates tested
☐	☒	A description of any assumptions or corrections, such as tests of normality and adjustment for multiple comparisons
☐	☒	A full description of the statistical parameters including central tendency (e.g. means) or other basic estimates (e.g. regression coefficient) AND variation (e.g. standard deviation) or associated estimates of uncertainty (e.g. confidence intervals)
☐	☒	For null hypothesis testing, the test statistic (e.g. *F*, *t*, *r*) with confidence intervals, effect sizes, degrees of freedom and *P* value noted<br>*Give P values as exact values whenever suitable.*
☒	☐	For Bayesian analysis, information on the choice of priors and Markov chain Monte Carlo settings
☒	☐	For hierarchical and complex designs, identification of the appropriate level for tests and full reporting of outcomes
☐	☒	Estimates of effect sizes (e.g. Cohen's *d*, Pearson's *r*), indicating how they were calculated

Our web collection on statistics for biologists contains articles on many of the points above.

Software and code

Policy information about availability of computer code

Data collection

Genomic and transciptomic analyses of patient samples: FASTQ_GENERATOR (bcl2fastq 2.19.0.316 and bcl2fastq 2.20.0.422), SEQUENCING_SOFTWARE (HCS 1.6.0, HCS 1.7.5, HCS 2.2.58, HCS HD 3.4.0.38, and HCS HD 3.5.0.7), BASECALL_SOFTWARE (RTA 1.18.64, RTA 2.7.7, and RTA v3.4.4).

DNA methylation arrays were scanned with iScan Control Software version 3.3.29.

RNA sequencing of cell lines: FASTQ_GENERATOR (bcl2fastq 2.20.0.422), SEQUENCING_SOFTWARE (HCS 2.2.58), BASECALL_SOFTWARE (RTA 1.18.64).

ACT-seq: FASTQ_GENERATOR (bcl2fastq 2.20.0.422), SEQUENCING_SOFTWARE (HCS 4.0.1.41 and HCS 4.0.2.7), BASECALL_SOFTWARE (RTA 2.11.3).

Quantitative RT-PCR data were acquired with Bio-Rad CFXMaestro version 4.02325.418 to operate a C1000 Touch Thermal Cycler (BioRad).

Immunofluorescence or phase contrast images were acquired using Gen5 version 3.10 and Leica Application Suite X version 3.5.7.23225 softwares to operate a Lionheart FX automated microscope or a TCS SP8 confocal microscope (Leica), respectively.

Immunoblot images were taken with the Amersham Imager 600 version 2.0.0 software to operate an Amersham Imager 600 (GE Healthcare) or with Image Studio version 5.2 to operate a LI-COR Odyssey CLx system.

Anchorage-independent growth assays were scanned with Gen5 version 3.10 to operate a Lionheart FX automated microscope.

Luminescence was quantified with EnVision Workstation version 1.14.3049.1193 to operate an EnVision Multimode Microplate Reader (PerkinElmer).

Flow cytometry data were acquired with BD FACSDiva version 9.2 to operate a FACSCelesta (BD Bioscience).

Collection of mass spectrometry data involved the softwares Xcalibur version 4.6.67.17 (conrols measurement), Thermo Scientific SII version 17.0.468 (LC), and Orbitrap Exploris 480 version 4.1.335.19 (MS).

Data analysis

Analysis of RNA-seq patient data: t-SNE analysis of RNA-seq data was performed with the R package Rtsne version 0.13.The function removeBatchEffect from the limma package was used to mitigate contamination of the expression profiles of lung metastases by those of surrounding normal lung tissue. Genes differentially expressed between FUS/EWSR1-TFCP2 sarcoma and all other RMS subtypes were calculated using DESeq2 version 1.18.1. KEGG pathway and GOTERM analyses were performed with DAVID (https://david.ncifcrf.gov/tools.jsp)

DNA methylation analysis: Raw data (idat files) were used to run the sarcoma classifier version 12.2. For further analysis, data were processed using ssNoob from the minfi R package . Unreliable probes, cross-reactive probes, probes mapping to sex chromosomes, and probes overlapping with single-nucleotide polymorphisms were filtered out based on dbSNP version 150. Beta values were used for further analyses. t-SNE clustering was conducted using the M3C and the Rtsne R packages. Mean beta values were used for hierarchical clustering using Manhattan distance and Ward.D2 linkage. Correlation analysis on mean beta values was performed using Spearman correlation.

Analysis of RNA-seq data of cell lines: Samples were de-multiplexed and aligned to the 1000 Genomes Phase 2 assembly of the human reference genome (hs37d5) using STAR aligner version 2.5.3a. Duplicate reads were marked by the markdup module of sambamba version 0.6.5. The expression of genes annotated in the GENCODE version 19 gene model was quantified by the featureCounts utility of the subread package version 1.5.1. RNA-SeQC version 1.1.8 was used to confirm data quality. A batch effect observed in MCF10A cells affecting the first replicate of each condition was corrected using the removeBatchEffect function of the limma package version 3.34.6. Read counts were normalized to the sample sequencing depth and transformed using the variance-stabilizing transformation method of the DESeq2 package version 1.18.1.

Analysis of ACT-seq data: Raw sequencing data were processed with the ChIP-Seq narrow peak version 1.2.1 and ATAC-Seq broad peak version 1.2.1 pipelines of the nf-core framework (14) to detect transcription factor binding sites and histone modifications, respectively. Differential peaks were called using the edgeR module of DiffBind v2.16.2, correcting for antibody- and batch-specific confounding effects whenever samples clustered by antibody or batch in principal component analysis. Enrichment of binding motifs inside ACT-seq peaks was determined with HOMER version 4.11 using peaks from EV controls as background and a fixed peak size of 1,000 base pairs. Visualizations of coverage peaks were generated with Integrative Genomics Viewer version 2.12.3.

Predictions of ALK protein structures: TThe complete structure for human ALK-WT has been predicted by AlphaFold and is publicly available (https://alphafold.ebi.ac.uk/entry/Q9UM73). While the single domain predictions seemed to be reasonable, the overall structure was problematic with respect to the placement of the domains in three-dimensional space. For instance, the kinase domain is closely placed with the extracellular domains, which is not a valid topology. Indeed, AlphaFold does not account for transmembrane proteins, and hence the predicted structures for such proteins can be topologically inaccurate. Thus, we used the HHpred server with PDB and Pfam as target databases. After the initial search, which yielded significant hits in the two MAM domains, the LDL receptor class A, the extracellular region (ECR), and the tyrosine kinase domain, the structure of the short LDL receptor was predicted using models constructed with manually edited/extended alignments from HHpred and with MODELLER. The approximate locations of the signal peptide and the transmembrane helix were determined using Phobius. For predicted structural regions, the corresponding PDB files were downloaded and processed using PyMOL version 2.0. To predict the domain structure of ALK variants, the HHpred server was used with Pfam as the target database. After the initial search, which yielded the approximate location of the MAM domains, the glycine-rich region of the ECR, and the kinase domain, BLAST alignments of ALK-WT and the five ALK variants were generated to determine the location of the EGF-like domain, which is part of the ECR, in the variants. For each variant, the presence of a signal peptide and a transmembrane helix was determined using Phobius.

Mass spectrometry: DIA raw files were converted to the mzML (32bit) format via MSConvert (3.0.21048) selecting ‘TPP compatibility’, ‘Write index’ and using the filter ‘peak picking’ and ‘title maker’. mzML files were analyzed via DIA-NN (version 1.8). The analysis was performed under the default parameters with the following changes: The number of allowed missed cleavages was set to 2 for Trypsin/P, ‘N-Term M excision’ and ‘Carbamidomethylation’ were selected, peptide length was 7-30 amino acids, ‘Mass accuracy‘ and ‘MS1 accuracy’ was set to 0 (automatic), ‘use isotopologues’ and ‘match between runs (MBR)’ was enabled, neuronal network classifier was set to ‘Double-pass mode’, protein inference was ‘Protein names from FASTA’, and ‘Optimal results’ was chosen for speed and RAM usage. The data was searched using an in-silico predicted spectral library (created by DIA-NN with smart profiling) of the human reference proteome from Uniprot with the sequence of ALK-ST4 added (containing 42,433 unique entries from July 18, 2023).

Colony formation assay images were quantified with ImageJ version 1.53e and the ColonyArea macro.

Anchorage-independent growth assays were counted using Gen5 software version 3.10 (Bio-Tek).

gH2AX foci quantification was performed using CellProfiler software version 4.1.3 (https://cellprofiler.org).

Nuclear fusion index from immunofluorescence images was determined with ImageJ version 1.53e.

Flow cytometry data were analyzed with FlowJo version 10.7.1.

For manuscripts utilizing custom algorithms or software that are central to the research but not yet described in published literature, software must be made available to editors and reviewers. We strongly encourage code deposition in a community repository (e.g. GitHub). See the Nature Portfolio guidelines for submitting code & software for further information.

Data

Policy information about availability of data

All manuscripts must include a data availability statement. This statement should provide the following information, where applicable:

- Accession codes, unique identifiers, or web links for publicly available datasets
- A description of any restrictions on data availability
- For clinical datasets or third party data, please ensure that the statement adheres to our policy

Sequencing and DNA methylation data from TFCP2-rearranged patient samples have been deposited in the European Genome-Phenome Archive (EGA) under accession code EGAS00001006939 [https://ega-archive.org/studies/EGAS00001006939]. Sequencing data from Horak et al.22 are available under accession code EGAS00001004813 [https://www.ebi.ac.uk/ega/studies/EGAS00001004813]. All data deposited under accession codes EGAS00001006939 and EGAS00001004813 are available under restricted access [https://ega-archive.org/dacs/EGAC00001000452]. The RNA-seq and ACT-seq data from cell lines generated in this study have been deposited in the Gene Expression Omnibus under accession code GSE224183 [https://www.ncbi.nlm.nih.gov/geo/query/acc.cgi?acc=GSE224183] and are publicly available. The mass spectrometry data for the detection of ALK-ST4 have been deposited in the ProteomeXchange Consortium via the PRIDE63 partner repository with the dataset identifier PXD045522 [http://www.ebi.ac.uk/pride/archive/projects/PXD045522] and are publicly available. The deposited data contain raw sequencing, methylation, or mass spectrometry data. Source data including raw values and uncropped western blot scans are provided with this paper.

Research involving human participants, their data, or biological material

Policy information about studies with human participants or human data. See also policy information about sex, gender (identity/presentation), and sexual orientation and race, ethnicity and racism.

Reporting on sex and gender	The sex of the 12 patients with FUS/EWSR1-TFCP2 fusions is indicated in Supplementary Data 1, demonstrating that the male-to-female ratio in this sarcoma subtype is 1. The data were collected within the MASTER and INFORM studies.We obtained consent from all patients to publish information that could be used to identify individuals.
Reporting on race, ethnicity, or other socially relevant groupings	We did not consider race, ethnicity, or other socially relevant factors in this study.
Population characteristics	The characteristics of the 12 patients with FUS/EWSR1-TFCP2 fusions are indicated in Supplementary Data 1. The additional data used from patients enrolled in the MASTER study have been reported before (Horak P, et al. Cancer Discov. 2021 Nov;11(11):2780-2795; doi: 10.1158/2159-8290.CD-21-0126.)
Recruitment	Patient recruitment into the MASTER and INFORM programs has been described previously in Horak et al., Cancer Discov. 2021 Nov;11(11):2780-2795 and van Tilburg et al., Cancer Discov. 2021 Nov;11(11):2764-2779, respectively.<br>Inclusion criteria of MASTER were as follows (text taken from Horak et al., Cancer Discov. 2021): "NCT/DKTK MASTER is a prospective, continuously recruiting, multicenter observational study for biology-driven stratification of adults with advanced cancer across histologies who are younger than 51 and patients with rare tumors, including rare subtypes of more common entities, regardless of age. In keeping with the therapeutic intent of the study, patients must have exhausted curative treatment options, be in good general condition (Eastern Cooperative Oncology Group performance status of 0 or 1), and provide written informed consent for banking of tumor and control tissue, molecular analysis, and the collection of clinical data under a protocol (S-206/2011) approved by the Ethics Committee of the Medical Faculty of Heidelberg University. The study was conducted in accordance with the Declaration of Helsinki."<br>INFORM is a prospective, noninterventional, multicenter, multinational, and feasibility registry collecting clinical, functional, and molecular data. This sentence was taken from van Tilburg et al., Cancer Discov. 2021. More detailed inclusion criteria can be found in the corresponding publication.
Ethics oversight	MASTER: The study was performed under a "protocol (S-206/2011) approved by the Ethics Committee of the Medical Faculty of Heidelberg University. The study was conducted in accordance with the Declaration of Helsinki." (Horak et al., Cancer Discov. 2021 Nov;11(11):2780-2795).<br>INFORM: "Approvals for the study protocol (and any modifications thereof) were obtained from independent ethics committees and the institutional review board at each participating center. The study was registered with the German Clinical Trial Register, number DRKS00007623." (van Tilburg et al., Cancer Discov. 2021 Nov;11(11):2764-2779)

Note that full information on the approval of the study protocol must also be provided in the manuscript.

Field-specific reporting

Please select the one below that is the best fit for your research. If you are not sure, read the appropriate sections before making your selection.

☒ Life sciences ☐ Behavioural & social sciences ☐ Ecological, evolutionary & environmental sciences

For a reference copy of the document with all sections, see nature.com/documents/nr-reporting-summary-flat.pdf

Life sciences study design

All studies must disclose on these points even when the disclosure is negative.

Sample size	No statistical method was used to predetermine sample size.<br>The sample size of data from primary patient samples was determined by sample/data availability.<br>For some in vitro analyses, three cell lines of different tissue backgrounds (MCF10A, SCP-1, and LHCN-M2) were used to determine effects in epithelial, mesenchymal, and myoblast cells, respectively. The remaining experiments were performed mainly in MCF10A cells. Cell line experiments were performed in three technical replicates and three biological replicates unless specified otherwise (see also Source Data file).<br>Xenotransplantations were carried out as a hypothesis-generating experiment in which no further specified hypotheses are to be tested. Three mice per group with two tumors each were used (n=6 tumors per cell line).
Data exclusions	No data were excluded.
Replication	Whole-genome and exome sequencing, RNA sequencing, and DNA methylation analysis of patient samples were performed once per sample as is common practice. RNA sequencing of cell lines was performed in at least three biological replicates. ACT-seq of cell lines was performed in three or four replicates (see Methods section). Functional analyses were performed in three independend experiments unless specified otherwise. All attempts at replication were successful.
Randomization	No randomization was performed. MASTER and INFORM are prospective observational studies. Randomization for experiments with cell lines and mice was not applicable.
Blinding	Investigators were not blinded to allocation and outcome assessment. MASTER and INFORM are prospective observational studies. Strict blinding of laboratory experiments was not possible since the identity of cell lines needed to be indicated on the culture flasks etc.

Reporting for specific materials, systems and methods

We require information from authors about some types of materials, experimental systems and methods used in many studies. Here, indicate whether each material, system or method listed is relevant to your study. If you are not sure if a list item applies to your research, read the appropriate section before selecting a response.

Materials & experimental systems

n/a	Involved in the study	
☐	☒	Antibodies
☐	☒	Eukaryotic cell lines
☒	☐	Palaeontology and archaeology
☐	☒	Animals and other organisms
☐	☒	Clinical data
☒	☐	Dual use research of concern
☒	☐	Plants

Methods

n/a	Involved in the study	
☒	☐	ChIP-seq
☐	☒	Flow cytometry
☒	☐	MRI-based neuroimaging

Antibodies

Antibodies used	Immunoblotting primary:<br>ALK (D5F3) XP, rabbit, 1:2,000 in 5% milk/TBST, Cell Signaling, Cat: 3633, Lot: 9812<br>Anti-β-Actin (AC-15), mouse, 1:5,000 in 5% milk/TBST, Sigma-Aldrich, Cat: A1978, Lot: 088M4804V<br>β-Tubulin, rabbit, 1:1,000 in 5% BSA/TBST, Cell Signaling, Cat: 2146, Lot: 9<br>EWS (G-5), mouse, 1:100 in 5% milk/TBST, Santa Cruz, Cat: sc-28327, Lot: L1917<br>Anti-FUS, rabbit, 1:500 in 5% milk/TBST, Sigma-Aldrich, Cat: SAB2108528, Lot: QC49718<br>GAPDH (D16H11), rabbit, 1:1,000 in 5% milk/TBST, Cell Signaling, Cat: 5174, Lot: 8<br>TFCP2 (D1S3V), rabbit, 1:1,000 in 5% BSA /TBST, Cell Signaling, Cat: 80784, Lot: 1<br>p44/42 MAPK (ERK1/2) (L34F12), mouse, 1:2,000 in 5% BSA/TBST, Cell Signaling, Cat: 4696, Lot: 22<br>Phospho-p44/42 MAPK (ERK1/2) (Thr202/Tyr204) (20G11), rabbit, 1:1,000 in 5% BSA/TBST, Cell Signaling, Cat: 4376, Lot: 18<br>Akt (pan) (40D4), mouse, 1:2,000 in 5% BSA/TBST, Cell Signaling, Cat: 2920, Lot: 8<br>Phospho-Akt (Ser473) (D9E) XP, rabbit, 1:2,000 in 5% BSA/TBST, Cell Signaling, Cat: 4060, Lot: 25<br>Phospho-Akt (Thr308) (244F9), rabbit, 1:1,000 in 5% BSA/TBST, Cell Signaling, Cat: 4056, Lot: 23<br>Stat3 (124H6), mouse, 1:1,000 in 5% BSA/TBST, Cell Signaling, Cat: 9139, Lot: 12<br>Phospho-Stat3 (Tyr705), rabbit, 1:1,000 in 5% BSA/TBST, Cell Signaling, Cat: 9131, Lot: 30<br>Recombinant Anti-Telomerase reverse transcriptase antibody (Tyr182), rabbit, 1:1,000 5% milk/TBST, Abcam, Cat: ab32020, Lot: 1041820-5<br><br>Immunoblotting secondary:<br>Goat Anti-Rabbit IgG H&L (HRP), Rabbit, 1:10,000 in 5% BSA/TBST, Abcam, Cat: ab6721, Lot: GR3357864-7<br>Rabbit Anti-Mouse IgG H&L (HRP), Mouse, 1:10,000 in 5% BSA/TBST, Abcam, Cat: ab6728, Lot: GR83817-10<br>Anti-rabbit IgG (H+L) (DyLight 680 Conjugate), Rabbit, 1:10,000 in 5% milk/TBST, Cell Signaling, Cat: 5366s, Lot: 7 and 9<br>Anti-mouse IgG (H+L) (DyLigh 800 4X PEG Conjugate), Mouse, 1:10,000 in 5% milk/TBST, Cell Signaling, Cat: 5257s, Lot: 7 and 8

Immunohistochemistry:
Desmin (5µg/ml, CC1 pretreatment, Roche/Ventana, Cat: DE-R-11, Lot: G12069)
MyoD1 (0,92 µg/ml, CC1 pretreatment, Cell Marque, Cat: EP212, Lot: V0001802)
Myogenin (212,2 mg/l, CC1 pretreatment, Cell Marque, Cat: F5D, Lot: 10112204)
ALK1 (57mg/l, CC1 pretreatment, DAKO, Clone ALK1, Lot: 20044563)
p44/42-(Thr202/Tyr204)-MAPK (p-ERK1/2; 502µg/ml, CC1 pretreatment, Cell Signaling, clone D13.14.4E, Cat: 4370, Lot: 28)
p-(Ser643/676)-PKCδ/θ (15µg/ml, CC1 pretreatment, Cell Signaling, Cat: 9376, Lot: 6)
p-Stat3 (Tyr705) (1:50, CC1 pretreatment, Cell Signaling, clone D3A7, Cat: 9145, Lot: 43)
p-(Ser473)-AKT (91µg/ml, CC1 pretreatment, Cell Signaling, clone D9E, Cat: 4060, Lot: 27).

Immunofluorescence (primary and secondary):
ALK, Cell Signaling Technology, D5F3, Cat: 3633, Lot: 9812
E-cadherin, Invitrogen, Cat: 13-1700, Lot: UF2761472A
Anti-MyoHC antibody, Thermo Fisher, Cat: 14-6503-82
Goat anti-rabbit IgG Alexa Fluor Plus 488, Invitrogen, Cat: A11008, Lot: 1348202 and 2521157
Goat anti-mouse IgG Alexa Fluor 633, Invitrogen, Cat: A-21052, Lot: 1010093
Alexa Fluor 488 mouse anti-H2AX (pS139) antibody, BD Bioscience, Cat: 560445, Lot: 1172555
HA Tag Alexa Fluor 647-conjugated antibody, R&D systems, Cat: IC6875R, Lot: AEUQ0320071

Flow cytometry:
Alexa Fluor 488 mouse anti-H2AX (pS139), BD Bioscience, Cat: 560445, Lot: 1172555
Alexa Fluor 647 mouse anti-HA Tag, R&D systems, Cat: IC6875R, Lot: AEUQ0320071

ACT-seq:
HA-Tag (C29F4) rabbit mAb, Cell Signaling, Cat: 3724, Lot: 9
HA-Tag (F-7) mouse mAb, Santa Cruz, Cat: sc-7392, Lot: F1621
Anti-histone H3 (acetyl K27) antibody – ChIP grade, Abcam, Cat: ab4729, Lot: GR3374555-1
Rabbit IgG polyclonal antibody, Merck, Cat: PP64

Validation

ALK antibody was validated by ALK variant overexpression and Western blotting (Supplementary Fig. 3a and i).
FUS, EWS, and TFCP2 antibodies were validated by overexpression of the respective genes and Western blotting (Supplementary Fig. 4).
Standard loading control antibodies for Western blotting were not validated (Anti-β-Actin, β-Tubulin, GAPDH).
Antibodies detecting signaling proteins ERK1/2, AKT, and STAT3 and their phosphorylation are widely used and validated for western blotting by the company (Cell Signaling).
Anti-TERT antibody (Recombinant Anti-Telomerase reverse transcriptase antibody (Tyr182)) is widely used and validated (see product page at Abcam).
Immunhistochemistry antibodies are used in pathology routine diagnostics.
E-cadherin antibody was validated by correct membrane staining in epithelial cells (Suppl. Fig. 3h).
Anti-histone H3 (acetyl K27) antibody used for ACT-seq is a widely used and a dedicated ChIP grade antibody.
Anti-MyoHC antibody is widely used and validated by the company (Invitrogen/Thermo Fisher Scientific); it is also validated by the specific typical staining pattern that increases during myogenic differentiation (Figure 4b, Supplementary Fig. 4g)
HA antibodies used in ACT-seq were validated by specific detection of HA-tagged TFCP2 fusion genes and the respective wildtype genes by Western blotting.
Alexa Fluor 488 mouse anti-H2AX (pS139) antibody was validated by the company (BD Bioscience) by flow cytometry and immunofluorescence.
HA Tag Alexa Fluor 647-conjugated antibody was validated by the company (R&D) by flow cytometry.

Eukaryotic cell lines

Policy information about cell lines and Sex and Gender in Research

Cell line source(s)

MCF10A were obtained from ATCC (Cat: CRL-10317)
SCP-1 were described previously (Trautmann M, et al., EMBO Molecular Medicine. 2019;e9889-15.)
LHCN-M2 were obtained from Evercyte (Cat: CkHT-040-231-2)
HEK293T are standard cells used for virus production, were kindly provided by William C. Hahn , and were previously described (e.g. Trautmann M, et al., EMBO Molecular Medicine. 2019;e9889-15.)

Authentication

Cell line identity was verified using the Multiplex Cell Authentication Test (Multiplexion) or the Human Cell Line Authentication Service (Eurofins Genomics Germany).

Mycoplasma contamination

All cell lines are routinely tested for mycoplasma contamination and were tested negative.

Commonly misidentified lines
(See ICLAC register)

The cell lines used are not listed in the ICLAC register.

Animals and other research organisms

Policy information about studies involving animals; ARRIVE guidelines recommended for reporting animal research, and Sex and Gender in Research

Laboratory animals

Xenotransplantations were perfomed with 7 to 8 week old NOD/SCIDIL2rgnull mice.
Mice were housed in the DKFZ Center for Preclinical Research. In accordance with the Appendix A of des European Convention for

the Protection of Vertebrate Animals used for Experimental and Other Scientific Purposes from 18th March 1986, room temperature and relative humidity were adjusted to 22.0 ± 2.0 °C and 55.0 ± 10.0 %, respectively. All animals were housed under strict specified pathogen-free (SPF) conditions according to the recommendations of the Federation of European Laboratory Animal Science Associations (FELASA). The light/dark (L/D) cycle was adjusted to 14 hours lights on and 10 hours lights off with the beginning of the light and dark period set at 6 am and 8 pm, respectively.

Wild animals

The study did not involve wild animals.

Reporting on sex

Female mice were used as they were available at the time of the experiment. The findings are not influenced by the sex.

Field-collected samples

The study did not involve samples collected from the field.

Ethics oversight

All animal procedures were approved by the regional authority in Karlsruhe, Germany (reference number 35-9185.81/G-75/16) and performed according to federal and institutional guidelines.

Note that full information on the approval of the study protocol must also be provided in the manuscript.

Clinical data

Policy information about clinical studies

All manuscripts should comply with the ICMJE guidelines for publication of clinical research and a completed CONSORT checklist must be included with all submissions.

Clinical trial registration

MASTER (ClinicalTrials.gov: NCT05852522) and INFORM (German Clinical Trial Registry: DRKS00007623) studies are non-interventional observational studies.

Study protocol

The complete study protocols are available upon request from the respective study center: MASTER: master@nct-heidelberg.de; INFORM: inform_info@dkfz.de).

Data collection

FUS/EWSR1-TFCP2-positive cases recruited until March 2022 were included in the study. In addition, data from patients enrolled in the MASTER study until November 21, 2018, were used (Horak et al., Cancer Discov. 2021 Nov;11(11):2780-2795).

Outcomes

A detailed description of outcome measures has been reported in the original publication of the MASTER study (Horak et al., Cancer Discov. 2021 Nov;11(11):2780-2795). Briefly, response was evaluated based on clinical and radiologic reports and categorized into five classes: complete response/no evidence of disease, partial response, mixed response (MR), stable disease for at least eight weeks, and progressive disease. MR, defined as concurrent response or stabilization of some and progression of other metastatic lesions accompanied by clinical benefit, was documented as an indication of biological drug efficacy and/or tumor heterogeneity. The outcomes of FUS/EWSR1-TFCP2 cases are presented in Supplementary Data 1.

Plants

Seed stocks

n/a

Novel plant genotypes

n/a

Authentication

n/a

Flow Cytometry

Plots

Confirm that:

☒ The axis labels state the marker and fluorochrome used (e.g. CD4-FITC).

☒ The axis scales are clearly visible. Include numbers along axes only for bottom left plot of group (a 'group' is an analysis of identical markers).

☒ All plots are contour plots with outliers or pseudocolor plots.

☒ A numerical value for number of cells or percentage (with statistics) is provided.

Methodology

Sample preparation

1x10*6 MCF10A cells stably transduced with EV, FUS-TFCP2, FUS, or TFCP2 were seeded in 75-cm2 flasks, and treatment was started the next day. Cells were either left untreated, incubated for four hours with 2.5 µg/ml cisplatin, or treated with 2.5 µg/ml cisplatin followed by cultivation in regular growth media for 24 hours to determine the effect of cisplatin release. Cells

were harvested, washed once with PBS, resuspended in 100 µl PBS, fixed and permeabilized by adding drop-wise 900 µl of ice-cold 100% methanol under gentle vortexing, and stored overnight at –20°C. Fixed cells were rehydrated by washing and incubation in 1 ml cold PBS overnight at 4°C. For staining, 0.5x10*6 cells were resuspended in 35 µl PBS containing 1% BSA and co-incubated with 5 µl Alexa Fluor 488 mouse anti-H2AX (pS139) antibody (BD Bioscience) and 5 µl Alexa Fluor 647 mouse anti-HA Tag (R&D systems) for one hour at 4°C in the dark. Cells were then washed, resuspended in 200 µl PBS containing 1% BSA, and acquired with a FACSCelesta (BD Bioscience). Data were analyzed with FlowJo v10.7.1 (BD Bioscience).

Instrument	FACSCelesta (BD Bioscience)
Software	FlowJo v10.7.1 (BD Bioscience)
Cell population abundance	No cell sorting was performed, only analysis.
Gating strategy	The main population of the fixed cells was gated in FSC and SSC. Then, doublets were excluded by gating on single cells in FSC-A and FSC-H. Next, HA-negative cells were gated for empty vector (EV) cells and HA-positive cells were gated for samples expressing HA-tagged FUS, TFCP2, or FUS-TFCP2. Finally, gH2AX-positive cells were determined. The gH2AX-positive gate was determined with untreated EV cells and applied to all samples.

☒ Tick this box to confirm that a figure exemplifying the gating strategy is provided in the Supplementary Information.
